# Finite Element Analysis of Anterior Implant-Supported Restorations with Different CAD-CAM Restorative Materials

**DOI:** 10.1055/s-0044-1785532

**Published:** 2024-05-14

**Authors:** Usanee Puengpaiboon, Nichapat Rattanapan, Vamsi Krishna Pasam, Chamaiporn Sukjamsri

**Affiliations:** 1 Department of General Dentistry, Faculty of Dentistry, Srinakharinwirot University, Bangkok, Thailand; 2 Department of Biomedical Engineering, Faculty of Engineering, Srinakharinwirot University, Nakhon Nayok, Thailand; 3 Department of Mechanical Engineering, National Institute of Technology Warangal, Warangal, Telangana, India

**Keywords:** dental implant, CAD-CAM crown materials, anterior teeth restoration, finite element analysis

## Abstract

**Objectives** Due to the lack of literature concerning the selection of crown materials for the restoration of anterior teeth, this study aimed to investigate the effects of six distinct computer-aided design and computer-aided manufacturing (CAD-CAM) crown materials on stress and strain distribution within implant-supported maxillary central incisor restorations, employing finite element analysis (FEA). Furthermore, a comparative analysis was conducted between models that incorporated adjacent natural teeth and those that did not, intending to guide the selection of the most suitable modeling approach.

**Materials and Methods** Crown materials, including Lava Ultimate, Enamic, Emax CAD, Suprinity, Celtra Duo, and Cercon xt ML, were the subjects of the investigation. FEA models incorporating Coulomb friction were developed. These models were subjected to an oblique load, simulating the average maximum bite force experienced by anterior teeth. The potential for failure in titanium implant components and the prosthesis crown was evaluated through von Mises and principal stress, respectively. Furthermore, the failure of crestal bone was assessed through principal strain values.

**Statistical Analysis** Stress values for each implant component and strain values of the bone were extracted from the models. To assess the impact of the six groups of crown materials, Kruskal–Wallis analysis of variance and post-hoc comparisons were conducted. Additionally, a statistical comparison between the two groups with Lava Ultimate and Cercon xt ML was performed using the Mann–Whitney U test to determine the difference in the two modeling approaches.

**Results** Higher crown material stiffness led to decreased stress in the abutment, fixture, and retaining screw, along with reduced strain in the surrounding bone. However, the decrease in stress and strain values became less significant with increasing crown stiffness. Additionally, the model with adjacent teeth showed significantly lower stress and strain concentrations compared to the model without adjacent teeth.

**Conclusion** Crowns with a high elastic modulus were the optimal choice for anterior teeth restoration. Constructing FEA models with adjacent teeth was highly recommended to gain a deeper understanding of the mechanical behavior of dental implant restorations.

## Introduction

Dental implants have become a well-established treatment for replacing missing teeth, thereby enhancing chewing function, oral health, and speech patterns. Notably, over 65% of implant restorations are performed for posterior teeth, particularly the first molars. 1 Although implant restoration for anterior teeth is less common, it is crucial and requires special attention due to the heightened aesthetic demand and patient expectations compared to posterior teeth.

Computer-aided design and computer-aided manufacturing (CAD-CAM) technology has become an invaluable tool in modern dental practice. Advancements in materials science and engineering have led to the development of various CAD-CAM restorative materials available in the form of blocks and discs. Presently, the ones with aesthetic features, that is, color and translucency level, close to the natural appearance of the anterior teeth, which may be used as a prosthetic crown for anterior implant restoration, include hybrid materials (Lava Ultimate, Enamic), glass-ceramics (E.max CAD, Suprinity, Celtra Duo), and 5Y-TZP zirconia (Cercon xt ML). 2 3 4

Due to variations in the elastic modulus of CAD-CAM materials, using different materials as prosthetic crowns in the same clinical scenario can lead to distinct mechanical effects. An inappropriate crown material may result in transmitted overload, causing excessive stress that can damage the implant components. It also can cause excessive or insufficient strain distribution in the surrounding bone, leading to crestal bone loss. 5 6 7 Selecting suitable crown materials is crucial for improving implant lifespan and the overall success of restorative treatment. However, the decision-making process regarding material choice may rely more on personal preferences and the expertise of the clinician than a comprehensive understanding of the mechanical characteristics of each material. 8 Since assessing the mechanical behavior of various materials is not a routine part of clinical practice, some clinicians may encounter challenges when determining the most suitable material, particularly for anterior implant restoration, which is not extensively documented in the literature.

Finite element analysis (FEA) is a powerful computational method used to predict the mechanical behavior of materials under loading, particularly those with complex shapes and properties. It is also reliable in providing accurate results in a wide range of dental implant applications. 9 Several FEA studies have explored the effects of crown materials on the mechanical performance of dental implants. However, these studies have primarily focused on posterior teeth restoration, and their findings have not been consistent as some studies reported that crown modulus was associated with stress concentration at specific locations, while others found no significant effect on certain implant components or bone. 10 11 12 13

It is well recognized that the characteristics of occlusal forces exerted on posterior and anterior teeth differ. In the case of posterior teeth, occlusal forces are typically greater in magnitude and primarily exerted along the long axis of the tooth. Conversely, for anterior teeth, the forces are comparatively smaller and tend to align obliquely with the tooth axis. Conducting an FEA specifically designed to simulate the masticatory behavior of anterior teeth would provide a more accurate depiction of the impact of crown material on anterior implant restoration.

To the best of the authors' knowledge, the majority of FEA studies conducted on dental implant restorations have utilized models that solely incorporated implant components and the surrounding bone, excluding adjacent teeth. Modeling in this way may not adequately represent the functional occlusion pattern. It is, therefore, important to determine whether the presence of adjacent natural teeth, along with their periodontal ligament (PDL), could potentially influence the outcomes.

This study aims to address the existing gaps in the literature regarding the effects of crown material on anterior teeth restorations and the reliability of the FEA modeling approaches. Two main objectives have been established for this research. The first objective is to employ FEA to investigate the influence of six different monolithic CAD-CAM crown materials, namely Lava Ultimate, Enamic, Emax CAD, Suprinity, Celtra Duo, and Cercon xt ML, on stress and strain distribution in implant components and the surrounding bone in the context of implant-supported maxillary central incisor restorations. The FEA models developed for this study incorporate adjacent teeth to simulate occlusion patterns. The second objective is to compare the FEA models with and without the inclusion of adjacent teeth to provide insights into the variations in stress and strain distribution resulting from the two modeling approaches. The findings of this study aim to provide clinicians confidence in selecting suitable crown materials for anterior teeth and constructing appropriate FEA models for further research in implant dentistry.

## Materials and Methods

**Fig. 1:**
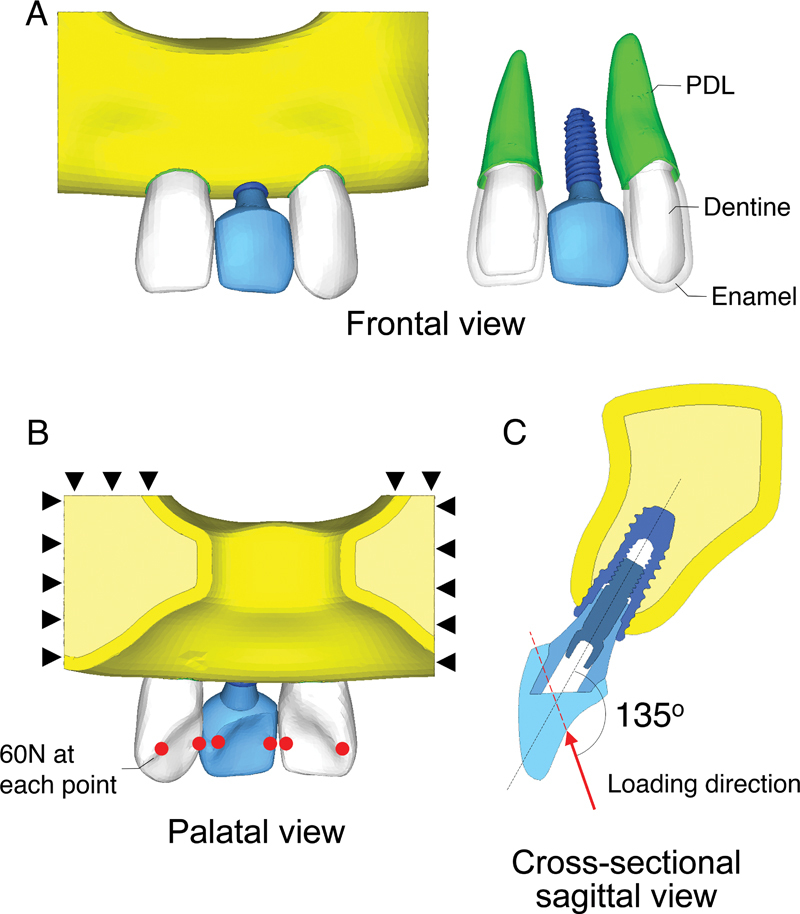
Finite element model of a maxillary left central incisor restored with a single implant-supported crown and the two adjacent teeth with periodontal ligament (PDL) ( **A** ). The black triangles indicate the superior and lateral borders of the bone, which are rigidly fixed to prevent motion, and the red dots represent the points of six positions of the load application, located at the middle of the mesial and distal marginal ridge ( **B** ). A simulated bite force of 60N was applied to each load application point, with a direction set at 135 degrees to the long axis of the tooth ( **C** ).

The first part of this study investigated the influence of crown materials. The three-dimensional (3D) model of a maxillary left central incisor restored with a single implant-supported crown and the two adjacent teeth with PDL was constructed ( Fig. 1A ). The bone and teeth models were modified from BodyParts3D (Database Center for Life Science, Tokyo, Japan). The internal structure of these models was created using Rhinoceros (Robert McNeel & Associates, Seattle, United States). The alveolar bone consisted of a cancellous bone region along with a cortical layer with a thickness of 1.6 mm. 14 The two adjacent teeth included a dentine region, an enamel layer with a thickness of 1 mm, 15 and PDL with a width of 0.2 mm. 16

The implant system, TSIII SA (Osstem Co., Seoul, Korea), was used in this study. The implant fixture, with an internal connection, had dimensions of 4 mm in diameter and 10 mm in length. Micro-computed tomography images of the fixture were obtained using a Skyscan 1172 scanner (Micro Phonics Inc., Pennsylvania, United States) and used to construct the 3D model of the fixture with Simpleware (Synopsys, Inc., California, United States). The 3D model of the prefabricated abutment with a retaining screw was created using Solidworks (Dassault Systems, Ile-De-France, France). A prosthetic crown matching the left central incisor was designed, and the assembled implant complex models were virtually placed in the truncated maxilla to represent immediate implant placement at the crestal cortical level.

Four-node linear tetrahedral elements were generated throughout the model using Simpleware. A mesh convergence test was performed successfully, with element sizes ranging from 0.3 to 1.2 mm. All materials were considered homogenous and isotropic, and the properties assigned to all components in this study were determined based on the literature ( Table 1 ). 2 17 18 19 20 21 22 23

**Table 1:** Material properties

Component		Material	Young's modulus (MPa)	Poisson's ratio
Crown	Lava Ultimate (3M ESPE)	Hybrid ceramic	11,050	0.368
	Enamic (VITA Zahnfabrik)	Hybrid ceramic	38,110	0.243
	E.max CAD (Ivoclar-Vivadent)	Glass-ceramics	102,800	0.214
	Suprinity (VITA Zahnfabrik)	Glass-ceramics	105,800	0.207
	Celtra Duo (Dentspy Detrey)	Glass-ceramics	108,200	0.224
	Cercon xt ML (Dentsply Sirona)	5Y-TZP zirconia	210,000	0.242
Fixture		CP Ti grade 4	110,000	0.34
Abutment		Ti-6Al-4V grade 5	115,000	0.34
Screw		Ti-6Al-4V grade 5	115,000	0.34
Cancellous bone			480	0.225
Cortical bone			11,776	0.35
Periodontal ligament			50	0.49
Dentine			16,000	0.25
Enamel			80,100	0.28

The Coulomb friction model was utilized. A friction coefficient of 0.5 was assigned to the abutment–fixture, retaining screw–fixture, and retaining screw–abutment interfaces, based on an experiment involving a titanium ball sliding on a titanium disc. 24 The implant fixture was assumed fully osseointegrated, and the restorative crown was assumed bonded to a titanium abutment with adhesive cement. Therefore, a glue contact model was used for the fixture–bone and crown–abutment interfaces. The superior and lateral borders of the maxillary bone were constrained to restrict motion in all degrees of freedom.

In the first step of FEA, a preload of 267N was defined for the retaining screw to replicate the tightening torque of 30 Ncm. 25 This torque level was recommended by the manufacturer. For the second step, a simulated oblique load of 60N was applied at each of the six midpoints on the mesial and distal marginal ridges of the teeth to replicate the average maximum bite force of 120N exerted on an individual anterior tooth in healthy adults ( Fig. 1B ). 26 The direction of the applied force was determined by the interincisal angle, which was set to 135 degrees to simulate a normal occlusion ( Fig. 1C ). 27 FEA process was conducted using HyperWorks (Altair Engineering, Inc., Michigan, United States).

The effects of crown material on stress distribution were assessed. Von Mises stress was selected to evaluate the implant fixture, retaining screw, and abutment, as they exhibit ductile behavior. Tensile (positive sign) and compressive (negative sign) principal stresses were employed to evaluate the prosthetic crown, considering its brittle nature. 28 29 For assessing the crestal bone, tensile and compressive principal strains were utilized. The first 50 most representative highest stress or strain values were collected for each implant component and the bone to statistically analyze the data. 30 31 The Shapiro–Wilk test was performed to assess data normality. Nonparametric Kruskal–Wallis analysis of variance was conducted, considering *p-* value less than 0.05 as statistically significant. Post-hoc comparisons were carried out using Dunn's test with Bonferroni correction. Statistical analysis was performed using Microsoft Excel with the Real Statistics Resource Pack software (Release 7.6). 32

**Fig. 2:**
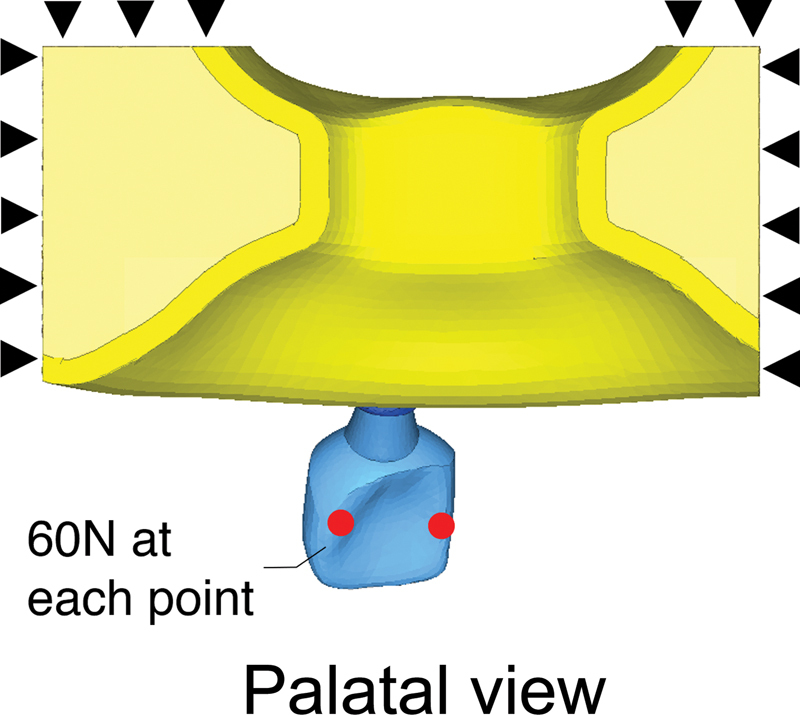
Finite element model without adjacent teeth. The black triangles indicate fixed boundary conditions. Red dots indicate loading points where a force of 60N is applied.

In the second part of this study, FEA models constructed for the first part were compared to those without the inclusion of adjacent natural teeth ( Fig. 2 ). Boundary conditions, loading direction, and magnitude followed the same simulation approach as in the first part. The models were assigned crown materials, namely Lava Ultimate and Cercon xt ML, representing the lowest and highest Young's modulus, respectively. The highest 50 values of stress and strain in the implant components and bone of both models were statistically compared using the Mann–Whitney U test.

## Results

**Fig. 3:**
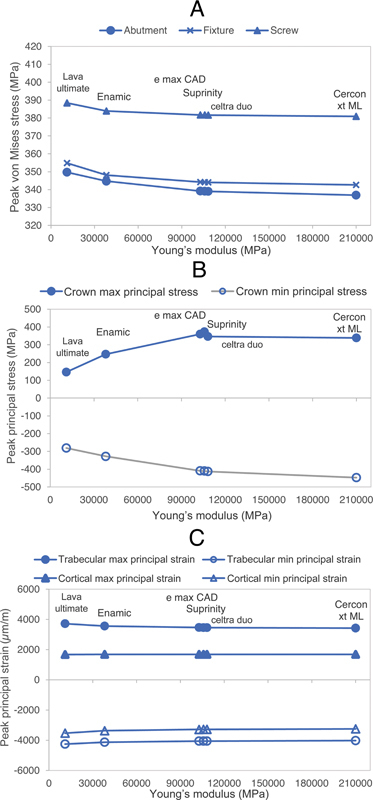
Scatter plots show the correlation between the peak von Mises stress at the implant component ( **A** ), peak principal stress at the crown ( **B** ), and peak principal strain in the trabecular and cortical bone ( **C** ), versus Young's modulus of different crown materials.

**Fig. 4:**
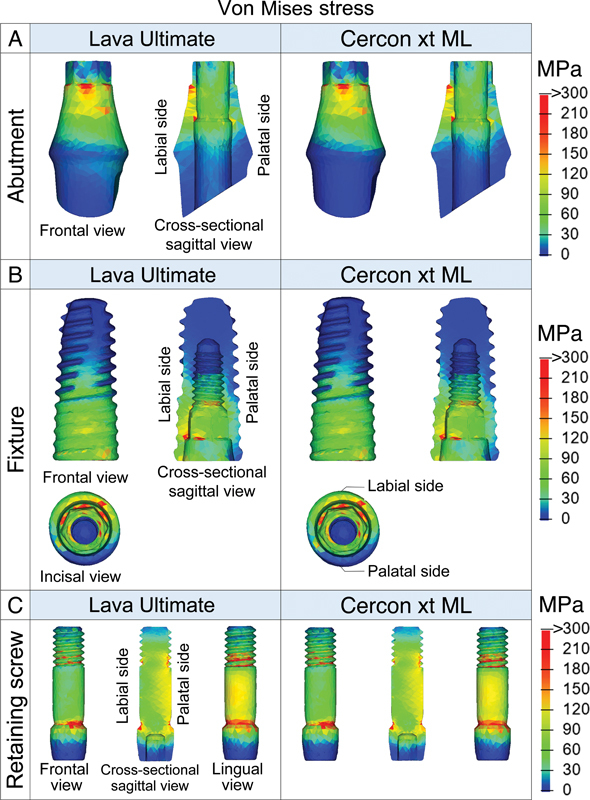
Comparison of von Mises stress distribution at the abutment ( **A** ), fixture ( **B** ), and retaining screw ( **C** ) between the model with an artificial crown made of Lava Ultimate (lowest Young's modulus) and Cercon xt ML crown (highest Young's modulus).

**Fig. 5:**
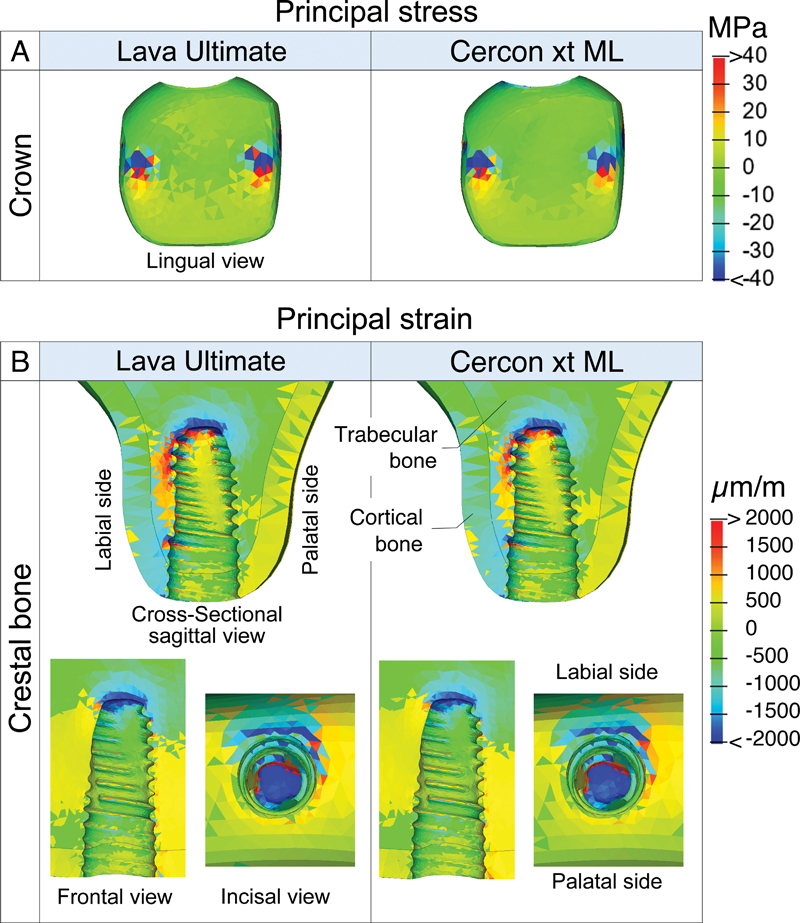
Comparison of principal stress distribution at the crown ( **A** ) and principal strain distribution at the trabecular and cortical bone ( **B** ), between the model with an artificial crown made of Lava Ultimate (lowest Young's modulus) and Cercon xt ML crown (highest Young's modulus). Positive signs indicate tensile stress or strain, while negative signs indicate compressive stress or strain.

The influence of crown material on the mechanical behavior of anterior implant-supported restorations is represented through scatter plots between peak stress (the highest among 50 values) at the implant components or peak strain at the crestal bone versus Young's modulus values of the crown material, shown in Fig. 3 . Statistical analysis, presented in Table 2 , determines the significance level of stress or strain variations compared among models with different crown materials. In Figs. 4 and 5 , stress and strain distribution patterns are compared between models utilizing the crown material with the lowest Young's modulus (Lava Ultimate) and the highest Young's modulus (Cercon xt ML).

**Table 2:** Statistical analysis with post-hoc comparison expressed by *p-* value (only statistic significant variables with Kruskal–Wallis analysis are reported)

Group 1	Group 2	Trabecular	Trabecular	Cortical
		Max principal strain	Min principal strain	Min principal strain
Lava Ultimate	Enamic	*p* < 0.05	0.09	0.14
Lava Ultimate	E.max CAD	*p* < 0.001	*p* < 0.05	*p* < 0.05
Lava Ultimate	Suprinity	*p* < 0.001	*p* < 0.05	*p* < 0.05
Lava Ultimate	Celtra Duo	*p* < 0.001	*p* < 0.05	*p* < 0.05
Lava Ultimate	Cercon xt ML	*p* < 0.001	*p* < 0.001	*p* < 0.05
Enamic	E.max CAD	0.12	0.29	0.33
Enamic	Suprinity	0.10	0.25	0.29
Enamic	Celtra Duo	0.08	0.22	0.26
Enamic	Cercon xt ML	*p* < 0.05	0.09	0.12
E.max CAD	Suprinity	0.93	0.93	0.94
E.max CAD	Celtra Duo	0.85	0.86	0.89
E.max CAD	Cercon xt ML	0.43	0.52	0.56
Suprinity	Celtra Duo	0.91	0.93	0.94
Suprinity	Cercon xt ML	0.48	0.57	0.61
Celtra Duo	Cercon xt ML	0.54	0.63	0.66

Note: Von Mises stress of the abutment, fixture, and retaining screw, maximum and minimum principal stress of the crown, and maximum principal strain of cortical bone, were not found to be significantly different following Kruskal–Wallis analysis.

### Stress in the Implant Components

For the three components considering ductile material, the highest range of von Mises stress was observed in the retaining screw followed by the fixture and abutment ( Fig. 3A ). Increasing the stiffness of the crown material resulted in a reduction in peak von Mises stress for all components. This relationship was not linear, with diminishing changes in peak stress as the Young's modulus of the crown material increased. Nevertheless, statistical analysis using the Kruskal–Wallis test revealed that different crown materials had no significant effect on stress in the retaining screw, fixture, and abutment. Stress patterns in the model with crown stiffness ranging from 11 GPa (Lava Ultimate) to 210 GPa (Cercon xt ML) were similar ( Fig. 4 ). Stress primarily concentrated at the implant–abutment connection near the implant platform, especially on the labial and palatal sides. The abutment and fixture showed greater stress on the labial side compared to the palatal side, while the retaining screw exhibited more concentrated stress on the palatal side over the labial side.

In the crown, stress mainly concentrates at the point of load application ( Fig. 5A ). Tensile and compressive stress increased with higher crown material stiffness ( Fig. 3B ). However, statistical analysis revealed no significant differences in tensile and compressive stress among models with different crown materials.

### Strain in the Crestal Bone

Tensile and compressive strain patterns remained unchanged with increased crown stiffness ( Fig. 5B ). Trabecular bone showed higher strain levels than cortical bone, with the highest strain near the apical end of the implant fixture. In the cortical bone, significant strain was observed on the labial side of the implant platform. Increased crown material stiffness led to slightly reduced peak tensile and compressive strains ( Fig. 3C ). Such finding was statistically significant for both tensile and compressive strain in the trabecular bone and compressive strain in the cortical bone ( Table 2 ).

### Comparison between Models with and without the Inclusion of Adjacent Natural Teeth

**Fig. 6:**
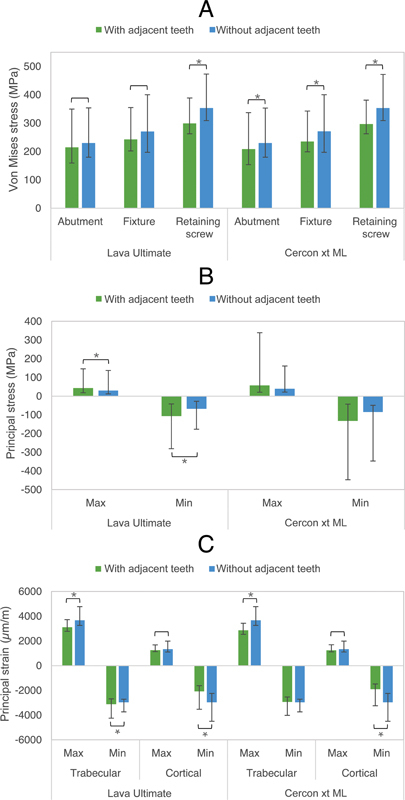
Comparison of von Mises stress ( **A** ), principal stress ( **B** ), and principal strain ( **C** ) between the model with and without adjacent teeth. Brackets indicate a significant difference with *p* -value less than 0.05. Brackets with an asterisk indicate significant differences with *p* -value less than 0.01.

Stress values at the implant components and strain values at the bone extracted from the model with and without adjacent teeth were compared and illustrated in Fig. 6 . The results indicated a significant difference at least with *p* -value less than 0.05 in von Mises stress at the abutment, fixture, and screw, between the model with and without the inclusion of adjacent teeth ( Fig. 6A ). Only for the Lava Ultimate group, a significant difference with *p-* value less than 0.001 was observed in principal stress at the crown ( Fig. 6B ). Furthermore, significant differences were observed in most principal strains at the trabecular and cortical bone. ( Fig. 6C ). A detailed statistical analysis using the Mann–Whitney U test is provided in Table 3 .

**Table 3:** Result of the statistical analysis in comparison between the model with and without adjacent teeth using the Mann–Whitney U test, expressed by *p-* value

		Lava Ultimate	Cercon xt ML
Abutment	Von Mises stress	<0.05	<0.001
Fixture		<0.05	<0.001
Screw		<0.001	<0.001
Crown	Max principal stress	<0.001	0.220
	Min principal stress	<0.001	0.053
Trabecular	Max principal strain	<0.001	<0.001
	Min principal strain	<0.001	0.099
Cortical	Max principal strain	<0.05	<0.05
	Min principal strain	<0.001	<0.001

## Discussion

This study employed a finite element model to explore the effects of crown material on the mechanical performance of a single implant-supported maxillary anterior restoration. The key findings indicate that higher crown material stiffness, characterized by Young's modulus, led to more favorable outcomes. Increasing crown stiffness reduced peak stress in the abutment, fixture, and retaining screw, as well as strain in the surrounding bone. However, only strain reduction was found to be statistically significant.

Comparing this study to previous research related to anterior teeth restoration is challenging due to limited literature. Although the influence of the elastic modulus of the crown on implant mechanics has been extensively studied for posterior teeth restoration, they still have been varied. Kaleli et al reported that an increased elastic modulus of the crown increased crown stress but did not affect stress in the abutment, implant fixture, and bone. 11 Tribst et al found that an increased elastic modulus of the crown reduced stress in the abutment but had no impact on the fixation screw and implant fixture. 12 Epifania et al concluded that the effect of crown material on the bone level is insignificant. 13 Additionally, Datte et al indicated that increased elastic modulus of crown materials reduced stress concentration in abutment and fixture with no differences in microstrain in the bone. 10 The variation in these results may be attributed to different clinical scenarios, including the focused group of crown material, implant design, and loading configuration. However, most of the mentioned studies agree that crown material with higher stiffness does not have adverse effects on implant components and bone, which corresponds to the findings of the present study. It can be explained that the masticatory forces exerting the implant and supporting bone are known to be transferred through the crown. If the crown material has a higher rigidity, the crown itself is less likely to deform. Consequently, the contact force transferred to the nearby component, which is the abutment, is reduced. The abutment and underlying structures, therefore, experience lower stress and the bone is less likely to deform under masticatory forces.

The increased stiffness of the crown material had no effect on the observed stress and strain patterns. In all models, von Mises stresses at the abutment, fixture, and retaining screw were concentrated at the implant–abutment connection region near the implant platform, which corresponds to the area where fractures primarily occur in real clinical scenarios. 33 34 The stress concentration was found on both the labial and palatal sides, which is related to the direction of the mastication force acting on the anterior teeth, causing the implant components to deform in tension at the palatal side and compression at the labial side. For all models, the maximum stresses observed at the implant system were found to be less than 390 MPa, which is notably lower than the reported strength of CP4–Ti (550 MPa) and Ti6Al4V (895–930 MPa), the materials from which the implant system is made. 35 This indicates that the implant system is unlikely to experience static failure under normal occlusal forces. Dental implant failure is primarily associated with cyclic loading, commonly known as fatigue. The reported fatigue limit of titanium dental implants is approximately 500 to 600 MPa. 35 36 Therefore, based on the findings of this study, it can be inferred that fractures are not expected to occur throughout the service life under normal occlusion. However, caution should be exercised when using restorative crowns with significantly lower rigidity than Lava Ultimate as materials with lower rigidity may increase the risk of crack initiation and potentially induce crown fractures, consequently reducing the treatment success. 7

Strain at the crestal bone is a crucial factor for predicting the long-term success of dental implants. According to the Frost mechanostat theory, microstrain range between 2,500 and 4,000 µm/m facilitates the stimulation of bone remodeling, while microstrain greater than 4,000 µm/m can induce internal crack formation that cannot naturally be repaired, potentially leading to implant disintegration. 37 In this study, the maximum compression strain observed in the trabecular bone around the apical region of the fixture measured approximately 4,300 µm/m, surpassing the optimum limit. However, the proportion of bone volume with overstrain was small, and high strain conditions may occur only in a short moment during mastication. For these reasons, the bone has the potential to undergo physiological adaptation, and hence permanent crack formation should not occur. 30

The FEA modeling approach in this study differs from most relevant literature as it accounts for the presence of adjacent teeth. A comparison between models with and without adjacent teeth revealed distinct differences, with the model featuring adjacent teeth exhibiting significantly smaller stress and strain concentrations. This outcome can be attributed to the allowance for contact between each tooth. The applied force of each tooth did not solely transfer to its supporting structures but was instead distributed over the region where its neighboring tooth was in contact. Additionally, the adjacent teeth included PDL, a thin layer with a very low elastic modulus, which helps absorb the transferred load. These two factors likely contribute to the reduction in transferred loads and subsequently lowered stress and strain concentrations in the implant components and surrounding bone compared to the traditional approach that may exaggerate the level of stress and strain concentration. In this study, abutment–fixture and abutment–screw interfaces were identified to allow microsliding following the prescribed coefficient of friction. In some previous studies, the contact counterparts in the abutment connection region were assumed to be perfectly bonded. 10 11 38 The latter, however, was more likely to demonstrate the monoblock implant, which was not a common system in the present dental market. Overall, this modeling approach provides a more realistic representation and better describes the mechanical behavior of dental implants under masticatory load.

Limitations of this study were addressed. First, it relied on a computational method, making it impractical to predict biological aspects such as bone remodeling and tissue response, which could have influenced the results. Nevertheless, several studies have validated the FE results through laboratory experiments and obtained accurate outcomes. 10 39 Second, the FE model used in this study was subjective in various aspects. It was constructed based on a specific oral structure, resulting in individual variations in teeth shape, bone structure, and implant orientation. Furthermore, this study focused on a specific implant design, which may differ from other studies. Therefore, the interpretation of the results necessitates careful consideration.

## Conclusion

The FEA study revealed that crown materials with varying stiffness levels had distinct effects on stress concentration. Crowns with high elastic modulus reduced stress concentration in implant components and minimized bone strain, making them suitable for anterior teeth restoration. The Cercon mt XL crown, with the highest elastic modulus, produced the best results in this study. The presence of adjacent teeth in the FE model significantly reduced stress and strain concentration compared to a model with only the restored tooth. Consequently, developing an FE model that includes the presence of adjacent teeth is highly recommended. It is important to note that this study was conducted in silico. Therefore, further studies should be undertaken, including experimental validation and clinical investigations.
